# Loneliness and cognitive aging: a brain-heart axis perspective on a modifiable risk

**DOI:** 10.3389/fragi.2026.1856056

**Published:** 2026-05-19

**Authors:** Alison Warren

**Affiliations:** 1 Department of Clinical Research and Leadership, George Washington University School of Medicine and Health Sciences, Washington, DC, United States; 2 Frame-Corr Laboratory, Washington, DC, United States; 3 Department of Continuing Education, Harvard University Extension School, Cambridge, MA, United States

**Keywords:** allostatic load, brain-heart axis, cardiovascular disease, cognitive aging, dementia, loneliness, social determinants of health, social isolation

## Abstract

Loneliness, the subjective perception of inadequate social connection, is increasingly recognized as a significant and modifiable determinant of health with implications extending beyond psychosocial well-being to cardiovascular and cognitive outcomes. A growing body of evidence links loneliness to increased risk of cardiovascular disease, cognitive decline, and dementia; however, these domains have often been studied in parallel. This review advances a brain–heart axis framework to integrate these literature, conceptualizing loneliness as a chronic psychosocial stressor that contributes to cognitive aging through interconnected neuroendocrine, autonomic, inflammatory, and vascular pathways. Loneliness is associated with sustained activation of stress-responsive systems, including the hypothalamic–pituitary–adrenal axis and sympathetic nervous system, leading to cumulative physiological burden, or allostatic load. These processes contribute to endothelial dysfunction, vascular injury, and increased risk of cardiovascular conditions, which in turn impair cerebral perfusion and promote neurodegeneration. In parallel, loneliness is associated with neurobiological effects, including hippocampal atrophy, neuroinflammation, and reduced cognitive reserve. Behavioral and psychological pathways further amplify these effects. This paper synthesizes epidemiologic, mechanistic, and clinical evidence to propose a multi-pathway model linking loneliness to cognitive aging through neurobiological and cardiovascular-mediated mechanisms, alongside behavioral and coping processes. Translational implications are discussed, positioning loneliness as a clinically actionable risk marker to support prevention and promote cognitive resilience in aging populations.

## 1 Introduction

Cardiovascular disease (CVD) and dementia are among the most pressing public health challenges worldwide (You, 2025). Population aging is reshaping global health systems and intensifying the burden of cognitive impairment and dementia. Worldwide, more than 55 million people are currently living with dementia, a number projected to exceed 139 million by 2050 (Alzheimer’s Disease International, 2025; WHO, 2025a). Dementia represents a leading cause of disability and dependency among older adults and poses substantial challenges for healthcare systems, families, and communities (World Health Organization, 2025). Despite advances in understanding Alzheimer’s disease and related dementias, disease-modifying therapies remain limited, prompting increasing emphasis on prevention and risk reduction across the life course (Livingston et al., 2024). The most recent Lancet Commission estimates that approximately 45% of dementia cases may be attributable to 14 modifiable risk factors, including cardiovascular, behavioral, and psychosocial exposures, such as depression, loneliness, and social isolation (Livingston et al., 2024). These findings emphasize the importance of identifying upstream determinants of brain health that operate well before the onset of clinical symptoms.

Within this evolving prevention landscape, loneliness has emerged as a significant and modifiable determinant of health (Warren, 2025). Loneliness involves subjective distress resulting from a perceived discrepancy between desired and actual social relationships (Perlman and Peplau, 1981; Perlman and Peplau, 1998). Although related to social isolation, loneliness represents a distinct psychological experience and may occur even in the presence of social networks (Hawkley and Cacioppo, 2010; National Academies of Sciences, Engineering, and Medicine, 2020). Epidemiologic evidence indicates that loneliness is prevalent across the life course, with increased vulnerability observed in midlife and older adulthood (Barreto et al., 2021; Hawkley, 2022).

A substantial body of research links loneliness to increased risk of morbidity and mortality, with effect sizes comparable to established behavioral risk factors such as smoking and obesity (Holt-Lunstad, 2021; Office of the Surgeon Gen eral OSG, 2023). In response, major public health organizations (e.g., the WHO, CDC, NASEM, U.S. Surgeon General, American Heart Association) have identified loneliness and social isolation as critical public health priorities requiring coordinated clinical and community responses (National Academies of Sciences, Engineering, and Medicine, 2020; WHO, 2025b; CDC, 2025; American Heart Association, 2025). Concurrently, loneliness has been linked to both CVD and cognitive decline; however, these domains have often been studied in parallel, with only recent frameworks beginning to integrate shared behavioral, psychological, and physiological mechanisms (Cené et al., 2022; Luchetti et al., 2024).

This review proposes a brain-heart axis framework to conceptualize loneliness as a chronic psychosocial stressor that contributes to cognitive aging through interconnected physiological systems. The aim of this paper is to integrate evidence across cardiovascular, neurological, and behavioral domains, and to provide a systems-level understanding of how loneliness becomes biologically embedded and to identify translational opportunities for clinical practice.

## 2 Loneliness as a cardiovascular and brain health exposure

Loneliness is increasingly recognized as a multidimensional exposure that influences health across psychological, behavioral, and biological domains (Warren, 2025). Conceptually, loneliness reflects perceived deficiencies in the quality or quantity of social relationships, whereas social isolation refers to the objective absence of social contacts (Perlman and Peplau, 1981; Hawkley and Cacioppo, 2010). Although related, these constructs may act through different mechanistic pathways (e.g., different inflammatory pathways) that contribute to adverse health outcomes, particularly in older populations (Cené et al., 2022; Sherman et al., 2024). This distinction is important for both research and clinical practice, as individuals may experience loneliness despite having social networks, while others may be socially isolated without perceiving loneliness.

Loneliness is shaped by a range of individual, relational, and structural factors, including socioeconomic disadvantage, life transitions such as retirement or bereavement, and broader patterns of social fragmentation (Mangot-Sala et al., 2025; Hutten et al., 2022). Prevalence estimates suggest that loneliness affects a substantial proportion of the population, with heightened vulnerability observed among older adults, individuals with chronic illness, and socially marginalized groups (Barreto et al., 2021; Hawkley, 2022). These patterns suggest that loneliness is not solely an individual-level phenomenon but a broader public health concern with implications for multiple domains of health.

A substantial body of epidemiologic research demonstrates that loneliness and social isolation are associated with increased risk of CVD (Cené et al., 2022; Sherman et al., 2024; Liang et al., 2023; Paul et al., 2021; Albasheer et al., 2024). Meta-analyses of longitudinal studies indicate that individuals experiencing loneliness have a significantly higher risk of coronary heart disease and stroke, with effect sizes comparable to traditional behavioral risk factors (Valtorta et al., 2016). Population-based cohort studies further demonstrate associations between loneliness and cardiovascular mortality, as well as incident conditions such as heart failure (Liang et al., 2023; Freak-Poli et al., 2021). Loneliness has also been linked to poorer outcomes among individuals with existing CVD, including reduced adherence to treatment, increased hospitalization, and diminished quality of life (Goodlin and Gottlieb, 2023).

Parallel to the cardiovascular literature, a growing body of research has established loneliness as a risk factor for cognitive decline and dementia. Longitudinal studies consistently demonstrate that individuals experiencing persistent loneliness exhibit accelerated decline in memory, executive function, and global cognition (Akhter‐Khan et al., 2021; Yu et al., 2023). A recent meta-analysis including more than 600,000 participants reported that loneliness is associated with a 31% increased risk of dementia (Luchetti et al., 2024). These findings persist after adjustment for depression, social isolation, and other confounders, suggesting that loneliness exerts independent effects on cognitive aging.

The sum of the evidence supports conceptualizing loneliness as a systems-level exposure that influences both cardiovascular and brain health. Rather than acting through a single pathway, loneliness appears to exert distributed effects across interconnected physiological systems, highlighting the need for integrative frameworks that bridge traditionally siloed domains of research.

## 3 The brain-heart axis: mechanistic framework

The brain-heart axis may provide a promising unifying framework for understanding how loneliness-related stress becomes biologically embedded across multiple systems. Implicated in other conditions of psychological distress, such as post-traumatic stress disorder, the brain–heart axis refers to an integrative pathway connecting frontal and limbic structures to the brainstem and peripheral organs via the autonomic nervous system (Seligowski et al., 2022). This axis represents a complex, bidirectional network integrating neural, cardiovascular, endocrine, and immune processes that collectively maintain physiological homeostasis (Scheitz et al., 2026; Valenza et al., 2025). Communication occurs through autonomic pathways, neuroendocrine signaling via the hypothalamic–pituitary–adrenal (HPA) axis, inflammatory mediators, and hemodynamic mechanisms influencing cerebral perfusion (Van Weperen et al., 2026). Importantly, this system operates bidirectionally: while the brain regulates cardiac function, cardiovascular states, including vascular integrity and cardiac output, also influence brain structure and function.

Within this framework, loneliness can be conceptualized as a chronic psychosocial stress exposure that disrupts brain-heart axis regulation. From an evolutionary perspective, perceived social isolation signals vulnerability, activating neural circuits involved in threat detection and promoting sustained vigilance (Hawkley and Cacioppo, 2010). While adaptive in acute contexts, persistent activation of these systems becomes maladaptive, contributing to chronic physiological dysregulation.

Loneliness has been associated with activation of stress-responsive pathways, including increased HPA-axis activity, elevated cortisol levels, and heightened sympathetic nervous system activation, alongside reduced parasympathetic tone (Cené et al., 2022; Xia and Loneliness, 2018). These changes contribute to allostatic load, defined as the cumulative physiological burden imposed by chronic stress across neuroendocrine, autonomic, immune, and metabolic systems (Warren, 2025; McEwen and Gianaros, 2011; McEwen, 2007). Over time, this cumulative burden leads to structural and functional changes across multiple organ systems, including endothelial dysfunction, arterial stiffness, reduced heart rate variability (Xia and Loneliness, 2018), and neurobiological alterations such as hippocampal atrophy (Dronse et al., 2023).

Chronic loneliness is also associated with low-grade systemic inflammation, reflected in elevated levels of inflammatory markers such as interleukin-6 and C-reactive protein (Warren, 2025; Cené et al., 2022). This inflammatory milieu contributes to vascular injury and atherosclerosis, while also influencing neuroinflammatory pathways and blood brain barrier (BBB) disruption implicated in cognitive decline (Cené et al., 2022; Anwer et al., 2026; Fahim et al., 2025). Autonomic dysregulation further contributes to impaired coordination between neural and cardiovascular systems, reinforcing a cycle of physiological dysregulation (Fahim et al., 2025; Rong et al., 2025).

Clinical manifestations of brain-heart axis dysregulation span a continuum from acute stress responses to chronic disease. Stress-induced cardiomyopathy (Takotsubo syndrome) provides a striking example of acute neurocardiac interaction, in which intense emotional stress triggers transient cardiac dysfunction (Singh et al., 2022). In contrast, chronic exposure to loneliness may contribute to long-term cardiovascular conditions such as myocardial infarction (Paul et al., 2021; Usama et al., 2024; Hakulinen et al., 2018) and heart failure (Goodlin and Gottlieb, 2023), which in turn influence cerebral perfusion and cognitive outcomes in older populations (Chen et al., 2022).

**FIGURE 1:**
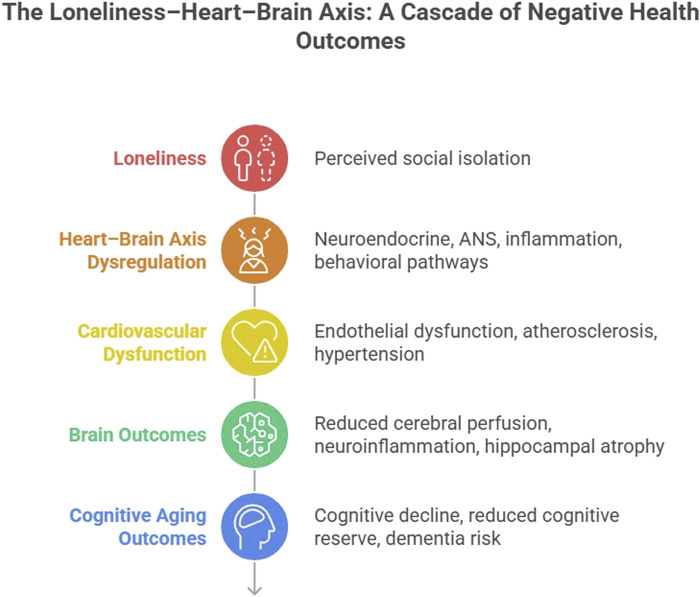
Loneliness–brain–heart axis conceptual model.

In sum, these processes support a model in which loneliness contributes to multisystem dysregulation within the brain-heart axis, linking psychological stress to both CVD and cognitive aging (see Figure 1 below).

Loneliness contributes to cognitive aging through interconnected neuroendocrine, autonomic nervous system (ANS), inflammatory, and behavioral pathways that drive dysregulation within the brain-heart axis. These processes promote cardiovascular dysfunction and neurobiological changes, including impaired cerebral perfusion and neuroinflammation, which interact bidirectionally to increase risk for cognitive decline and dementia. The model highlights key pathways and potential targets for clinical and translational intervention.

## 4 Loneliness, cardiovascular dysfunction, and cognitive aging

As cardiac and cognitive function are bidirectionally related (Park et al., 2024), CVD represents a major pathway linking loneliness to cognitive decline and *vice versa*. Cardiovascular conditions (e.g., stroke, heart failure, and atherosclerosis) contribute to cognitive impairment through mechanisms such as reduced cerebral perfusion, microvascular damage, and mixed vascular–neurodegenerative pathology (Livingston et al., 2024). Within this context, loneliness is particularly relevant because it is associated with increased risk of both CVD and cognitive decline. As previously mentioned, epidemiologic evidence demonstrates that loneliness is associated with increased risk of myocardial infarction, stroke, and heart failure (Liang et al., 2023; Valtorta et al., 2016). These conditions impair cerebral blood flow and contribute to cerebrovascular injury, which plays a critical role in cognitive decline.

In addition to overt cardiovascular and cerebrovascular events, a further plausible pathway by which loneliness may contribute to cognitive decline is through subclinical vascular brain injury, particularly cerebral small vessel disease (CSVD), which represents a key mechanistic link between cardiovascular pathology and neurodegeneration (Wardlaw et al., 2019). CSVD encompasses a spectrum of microvascular abnormalities, including white matter hyperintensities (WMH), lacunar infarcts, cerebral microbleeds, and enlarged perivascular spaces, which are highly prevalent in aging populations and strongly associated with both cardiovascular risk factors and cognitive impairment (Wardlaw et al., 2019; Debette and Markus, 2010).

**FIGURE 2:**
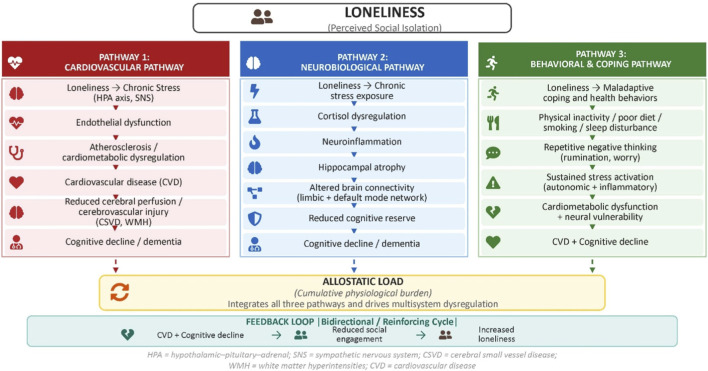
Multi-pathway model of loneliness within the brain–heart axis.

Among these markers, WMH are particularly well-established predictors of cognitive decline, executive dysfunction, and increased risk of dementia, including Alzheimer’s disease and vascular cognitive impairment (Nasiri et al., 2025; Luo et al., 2025). Emerging neuroimaging evidence suggests that loneliness may influence cognitive outcomes through this pathway, as WMH burden has been shown to mediate the association between loneliness and memory function, supporting a vascular mechanism linking psychosocial stress to brain aging (Park et al., 2023). Mechanistically, chronic loneliness-related stress may contribute to CSVD through endothelial dysfunction, impaired BBB integrity, and microvascular inflammation, processes that compromise cerebral perfusion and promote progressive white matter injury (Xia and Loneliness, 2018; Baumer et al., 2025). Furthermore, potential sex-specific heterogeneity may influence pathophysiological mechanisms underpinning loneliness and social isolation. For example, recent studies suggest sex-specific differences in the pathways linking loneliness and social isolation to vascular and cognitive outcomes. In older adults, loneliness was associated with memory dysfunction through white matter hyperintensity (WMH) burden in men but not in women, suggesting a stronger cerebrovascular mechanism underlying cognitive decline in males (Park et al., 2023). In contrast, social isolation has been linked to impaired endothelial function in women but not in men, indicating greater vascular sensitivity to psychosocial stressors among females and suggesting that sex-specific mechanisms may differentially influence cardiovascular and downstream cognitive risk (Sara et al., 2025). Cumulatively, the evidence suggests that loneliness contributes to cognitive decline not only through clinically apparent cardiovascular disease, but also through the gradual accumulation of subclinical vascular injury, highlighting a critical early intervention window for preventing both CVD and dementia. Further, this vascular pathway complements the neurobiological effects of loneliness, supporting a multi-level model in which psychosocial stress becomes biologically embedded through both neural and vascular mechanisms (Cené et al., 2022; Xia and Loneliness, 2018; Cacioppo and Hawkley, 2009). Loneliness may also influence cognitive aging through neurobiological pathways. Chronic activation of stress systems leads to dysregulations in cortisol, which have been linked to hippocampal atrophy and impaired memory function (Dronse et al., 2023). Resulting neuroinflammation and altered brain connectivity further contribute to cognitive vulnerability in this regard (Leng et al., 2023). Additional evidence further suggests that loneliness is associated with structural and functional brain changes in regions implicated in social cognition and memory, including reduced gray matter volume and altered connectivity within limbic and default mode networks, which may reflect the cumulative impact of chronic psychosocial stress on neural integrity (Costanzo et al., 2023). These neurobiological alterations are thought to interact with stress-related inflammatory and neuroendocrine processes, contributing to accelerated brain aging and increased vulnerability to neuropsychiatric disorders, cognitive decline, and dementia (Costanzo et al., 2023). These findings suggest that loneliness influences cognitive aging through both neurobiological pathways and cardiovascular-mediated pathways. (see Figure 2).

Importantly, these pathways are interconnected rather than independent. Cardiovascular dysfunction may exacerbate neural vulnerability, while central nervous system dysregulation may further impair cardiovascular function, creating a reinforcing cycle of risk. This dual-pathway model highlights the importance of integrating cardiovascular and neurological perspectives in understanding cognitive aging.

### 4.1 Behavioral pathways

Maladaptive health behaviors and coping mechanisms represent an additional pathway through which loneliness may contribute to both CVD and cognitive decline. Individuals experiencing loneliness are more likely to engage in behaviors such as physical inactivity, poor diet, smoking, and disrupted sleep, which are well-established risk factors for CVD and adverse cognitive outcomes (Cené et al., 2022; Paul et al., 2021; Loneliness, 2023; Shankar, 2023). Loneliness and its associated psychosocial stress have been correlated with cardiometabolic disruptions (e.g., hypertension, poor glucose control), weakened immunity, inflammatory responses, and mental health disorders (e.g., depression, anxiety, substance use, suicidality) (Paul et al., 2021; Usama et al., 2024). Loneliness has also been associated with reduced adherence to medical treatments and preventive health behaviors, further compounding cardiometabolic risk (Goodlin and Gottlieb, 2023; Hodgson et al., 2020). Sleep disturbance may serve as a critical intermediary, as insufficient or poor-quality sleep has been linked to hypertension, systemic inflammation, and impaired cognitive function (Hawkley, 2022; Paul et al., 2021). Concomitantly, loneliness may promote maladaptive psychological coping strategies, including rumination, hypervigilance to social threat, and negative cognitive bias, which sustain stress activation and reinforce behavioral risk patterns (Hawkley and Cacioppo, 2010; Cené et al., 2022; Paul et al., 2021). In this vein, repetitive negative thinking (RNT), including perseverative rumination and worry, has been identified as a key cognitive mechanism linking psychosocial stress to cardiovascular dysfunction, as it is associated with increased cardiac distress and dysregulated autonomic and physiological stress responses that may prolong stress system activation and contribute to cardiovascular risk (Sharif-Nia et al., 2025). Within this framework, loneliness-related rumination may function as a sustained cognitive stressor that exacerbates emotional distress and reinforces maladaptive psychophysiological responses, thereby amplifying both perceived and physiological burden (Sharif-Nia et al., 2025). These behavioral and psychological processes interact with underlying physiological mechanisms, amplifying allostatic load and contributing to cumulative vascular and neurobiological damage over time (Warren, 2025). This behavioral pathway may operate both independently and synergistically with cardiovascular and neurobiological pathways, reinforcing the role of loneliness as a multisystem exposure influencing cognitive aging through converging mechanisms (Paul et al., 2021). Together, these findings highlight the importance of considering health behaviors not merely as co-occurring risk factors, but as integral components of the pathways linking loneliness to cardiovascular dysfunction and cognitive aging.

### 4.2 Bidirectional and reverse causality within the brain-heart axis

While loneliness is a well-established risk factor for both cardiovascular disease and cognitive decline, the sum of available evidence supports a bidirectional relationship, in which cardiovascular and cognitive conditions may also increase vulnerability to loneliness (National Academies of Sciences, Engineering, and Medicine, 2020; Hakulinen et al., 2018; Karska et al., 2023). Individuals with cardiovascular disease frequently experience deterioration of overall health and physical functioning that diminishes their social participation, which can contribute to increased social isolation and perceived loneliness (Freak-Poli et al., 2021). Similarly, cognitive impairment and early dementia are associated with social withdrawal, communication difficulties, and reduced engagement in social activities, all of which may exacerbate loneliness and further compromise psychosocial wellbeing (Karska et al., 2023; Rehan et al., 2025).

Neurobiologically, structural and functional brain changes affecting social cognition, including alterations in limbic regions and the default mode network (Spreng et al., 2020), may further impair social perception and increase susceptibility to loneliness in individuals with cognitive decline. Within the brain–heart axis framework, the evidence collectively supports a reciprocal model (Scheitz et al., 2026) in which loneliness contributes to cardiovascular and cognitive dysfunction, while these conditions in turn reinforce loneliness, creating a self-perpetuating cycle that accelerates both physiological dysregulation and cognitive decline.

Loneliness contributes to cardiovascular disease (CVD) and cognitive decline through three interacting pathways. The cardiovascular pathway involves chronic stress–related activation of the hypothalamic–pituitary–adrenal (HPA) axis and sympathetic nervous system, leading to endothelial dysfunction, atherosclerosis, and cerebrovascular injury (e.g., cerebral small vessel disease), which impair cerebral perfusion and contribute to cognitive decline. The neurobiological pathway reflects effects of chronic stress on the brain, including cortisol dysregulation, neuroinflammation, hippocampal atrophy, and altered functional connectivity, resulting in reduced cognitive reserve. The behavioral and coping pathway includes maladaptive health behaviors (e.g., physical inactivity, poor diet, sleep disturbance) and repetitive negative thinking, which sustain stress activation and contribute to both cardiometabolic dysfunction and neural vulnerability. These pathways converge through allostatic load, representing cumulative physiological burden across systems. Bidirectional feedback loops illustrate how CVD and cognitive impairment may further increase vulnerability to loneliness, reinforcing a self-perpetuating cycle within the brain–heart axis.

## 5 Clinical translation across care settings

The convergence of evidence across cardiovascular and cognitive domains supports the integration of loneliness into clinical care as a modifiable risk factor (Warren, 2026). Loneliness is associated with increased risk of CVD, cognitive decline, and mortality, even after adjustment for traditional risk factors (Cené et al., 2022; Luchetti et al., 2024). This positions loneliness alongside other multidimensional risk factors, such as depression and frailty, that influence outcomes across disease states. Validated tools such as the UCLA Loneliness Scale enable assessment of loneliness in clinical settings (Russell et al., 1980; Mullen et al., 2019). However, implementation remains limited due to barriers including time constraints, lack of training, and uncertainty regarding intervention pathways (Mullen and Lum, 2025). Importantly, Evidence from social determinants of health screening indicates that screening alone may have limited impact, with improved outcomes observed when screening is paired with navigation or referral interventions (National Academies of Sciences, Engineering, and Medicine, 2020; Gottlieb et al., 2013).

Potential interventions include behavioral therapies, social prescribing, and community-based programs designed to enhance social connection (Galvez-Hernandez et al., 2022). Indeed, a growing body of evidence from systematic reviews and intervention studies supports the effectiveness of multidimensional approaches to reducing loneliness in older adults. A large systematic review and meta-analysis found that interventions targeting social connection, including group-based activities, psychological interventions, and social engagement strategies, are associated with reductions in loneliness in older adults, with greater effectiveness observed in interventions that are adaptable, theory-informed, and tailored to individual needs (Hoang et al., 2022).

Notably, cognitive-behavioral therapies (CBT) that specifically address maladaptive social cognitions have demonstrated the strongest evidence base among various populations, consistently outperforming approaches that focus solely on increasing social contact, (Eccles and Qualter, 2021; Masi et al., 2011). These interventions are grounded in the recognition that loneliness is often driven by distorted perceptions of social relationships and self-worth, requiring targeted cognitive restructuring rather than simple increases in social exposure. (Masi et al., 2011), thereby addressing major components of the psychological distress accompanied by the experience of loneliness, along with associated negative cognitive biases, hypervigilance to social threat, and maladaptive social expectations (Hawkley and Cacioppo, 2010). Meta-analytic evidence suggests that interventions targeting these maladaptive cognitions are among the most effective approaches for reducing loneliness in adults (Masi et al., 2011). A further example was illustrated by the LISTEN intervention, a combined CBT and narrative-based program, has been shown to reduce loneliness while also improving selected biological stress markers, psychosocial functioning, and quality of life among individuals with chronic illness (Theeke et al., 2016). These studies support the interconnected biological and psychosocial dimensions of loneliness and support the use of integrative intervention approaches. It is worth noting, however, that recent evidence indicates that the effectiveness of CBT-based approaches may vary across populations and intervention contexts. A systematic review and meta-analysis found that CBT-based interventions were less effective than reminiscence-based and social identity interventions among older adults, suggesting that cognitive approaches alone may not be universally optimal (Hickin et al., 2021; Cawthorne et al., 2022). The importance of considering population-specific factors, including age, cognitive status, and social context, when selecting interventions for loneliness may serve to optimize outcomes in this regard. In older populations, interventions that emphasize meaning-making, social identity, and life review may be particularly relevant, reflecting the broader psychosocial dimensions of loneliness in later life (Cawthorne et al., 2022).

Accordingly, there is increasing support for a modular and integrative approach to loneliness interventions, in which CBT-informed strategies are combined with other evidence-based components tailored to individual needs. Modular cognitive-behavioral frameworks allow for flexible targeting of key mechanisms, including maladaptive cognitions, social skills deficits, and behavioral avoidance, while incorporating complementary strategies such as social engagement and identity-based interventions (Cawthorne et al., 2022; Käll et al., 2020). This approach may be particularly appropriate given the heterogeneity of loneliness across the lifespan, including variability in underlying mechanisms, comorbid conditions, and social environments.

Taken together, the evidence suggests that while CBT represents a valuable component of loneliness interventions, optimal approaches are likely to be multimodal, combining cognitive, behavioral, and social strategies within a broader, context-sensitive framework. Integrating CBT-based approaches with social prescribing and community-based interventions may therefore provide a more comprehensive strategy that addresses both the psychological and structural dimensions of loneliness.

Team-based care models may facilitate implementation by integrating behavioral health, social work, and community resources into clinical care, particularly through emerging models that link clinical services with social care systems (National Academies of Sciences, Engineering, and Medicine, 2020; AHRQ, 2025; Beidler et al., 2023; Alderwick et al., 2018). These approaches align with broader efforts to address social determinants of health within healthcare systems.

## 6 Implementation, ethics, and equity

While public health policies to address loneliness have been offered (e.g., office of the Surgeon General), clinical integration of loneliness screening and intervention remains scarce despite best practice recommendations (Warren, 2026; Pollak et al., 2025). Integrating loneliness into clinical care raises important ethical considerations, particularly when intervention capacity is limited. Screening without the ability to provide meaningful support may create unmet expectations for patients and contribute to clinician moral distress (Garg et al., 2016). Ethical implementation therefore requires ensuring that screening is accompanied by clear pathways for action.

Loneliness is shaped by structural determinants, including socioeconomic disadvantage, social exclusion, and environmental factors (Mangot-Sala et al., 2025; Paul et al., 2021). These determinants intersect with health disparities in CVD and cognitive aging, highlighting the importance of equity-focused approaches (Ferguson et al., 2023). Addressing loneliness requires not only individual-level interventions but also broader structural strategies that enhance social infrastructure and access to resources.

## 7 Future research directions

Future research can prioritize advancing mechanistic understanding of the loneliness–brain-heart axis, including longitudinal studies examining causal pathways. Studies may also evaluate the extent to which CVD mediates the relationship between loneliness and cognitive decline. Intervention research is needed to determine whether reducing loneliness improves physiological markers and clinical outcomes. Translational and implementation science will be critical for bridging the gap between evidence and practice, particularly in advancing the integration of loneliness into routine clinical care. Frameworks such as the Knowledge-to-Action (KTA) process emphasize the importance of adapting evidence to local contexts, identifying barriers and facilitators, and iteratively evaluating implementation strategies (Graham et al., 2006). Similarly, implementation science models such as the Consolidated Framework for Implementation Research (CFIR) highlight the role of intervention characteristics, inner and outer setting factors, and processes in determining successful uptake within healthcare systems (Damschroder et al., 2009; Damschroder et al., 2022). Within this context, rigorous evaluation of screening strategies, care delivery models, and health system integration approaches will be essential to ensure that loneliness identification is both feasible and linked to actionable response pathways. In addition, the use of established implementation outcomes (including acceptability, appropriateness, and feasibility) provides a structured approach to assessing real-world impact and scalability (Proctor et al., 2015). Collectively, these approaches support the development of sustainable, equity-informed models that move beyond identification toward meaningful clinical and community integration. Economic evaluation and equity-focused research are also needed to inform scalable and sustainable interventions.

## 8 Conclusion

Loneliness is a significant and modifiable determinant of health that influences both cardiovascular and cognitive outcomes. Through interconnected pathways involving stress physiology, inflammation, vascular dysfunction, and behavioral processes, loneliness contributes to multisystem dysregulation within the brain-heart axis. Reframing loneliness as a biologically embedded exposure provides a foundation for integrating psychosocial and biomedical approaches to prevention. Addressing loneliness within clinical care and broader social systems represents a critical opportunity to promote cardiovascular health, enhance cognitive resilience, and support healthy aging.
